# A survey of Israeli physical therapists regarding reactive balance training

**DOI:** 10.1186/s12877-023-04356-5

**Published:** 2023-10-13

**Authors:** Noam Margalit, Ilan Kurz, Oren Wacht, Avril Mansfield, Itshak Melzer

**Affiliations:** 1 Schwartz Movement Analysis & Rehabilitation Laboratory, Department of Physical Therapy, Faculty of Health Sciences, Recanati School for Community Health Professions, Ben-Gurion University of the Negev, P.O.B. 653, 84105 Beer-Sheva, Israel; 2 Department of Emergency Medicine, Recanati School for Community Health Professions, Ben-Gurion University of the Negev, Ben-Gurion University, Beer-Sheva, Israel; 3 KITE-Toronto Rehabilitation Institute, University Health Network, Toronto, ON Canada; 4 Department of Physical Therapy, University of Toronto, Toronto, ON Canada; 5 Evaluative Clinical Sciences, Hurvitz Brain Sciences Program, Sunnybrook Research Institute, Toronto, ON Canada

**Keywords:** Accidental Falls, Reactive balance training, Physical Therapy, Survey, Knowledge translation, Barriers, Facilitators, Postural balance

## Abstract

**Background:**

‘Reactive balance training’ (RBT) was developed to improve balance reactions to unexpected losses of balance. Although this training method is effective, its practical usage in the field of physical-therapy in Israel and world-wide is still unclear.

**Aims:**

This study aimed to evaluate the extent of RBT use in physical-therapy clinics in Israel, to identify the significant barriers to/facilitators for implementing RBT in clinical practice among physical therapists, and to determine which aspects of RBT most interest physical therapists in Israel.

**Methods:**

Physical therapists in Israel completed a survey using a questionnaire regarding their knowledge and use of RBT in their clinical practices. We compared the specific use of RBT among users; non-users; and open-to-use physical therapists. The odds ratios of the facilitators and barriers were calculated using univariate and multivariate logistic regression models.

**Results:**

Four-hundred and two physical therapists responded to a yes/no question regarding their use of RBT. Three-quarters (75.4%) of physical therapists reported using RBT in their practices. The most prevalent barrier cited was insufficient space for setting up equipment and most prevalent facilitator was having a colleague who uses RBT. Most of the respondents wanted to learn more about RBT, and most of the non-users wanted to expand their knowledge and mastery of RBT principles.

**Conclusions:**

There are misconceptions and insufficient knowledge about RBT among physical therapists in Israel, indicating that they may falsely believe that RBT requires large and expensive equipment, suggesting they categorize RBT as external perturbation training only. Reliable information may help to improve general knowledge regarding RBT, and to facilitate the more widespread implementation of RBT as an effective fall-prevention intervention method.

**Supplementary Information:**

The online version contains supplementary material available at 10.1186/s12877-023-04356-5.

## Introduction

As life expectancy rises, there are more older adults who are at risk of falling. Older adults have sensorimotor deficits that may affect balance control [1–3], voluntary step abilities [4–6] and reactive balance control in standing and walking [7, 8], indicating a growing fall risk that may require intervention. Falls may have life-threatening consequences that can seriously affect the lives of older adults such as physical injuries [9, 10], psychological influences including fear of falling [11, 12] and serious financial burden [13]. In Israel, every day about 1000 older adults visit emergency departments due to a fall, and the total direct cost of hip fracture in the elderly population in Israel in 2013 was about 200 million USD [14].

Fall-prevention programs were found to be effective for reducing fall rates in older adults. Systematic reviews [15–17] that examined fall-prevention randomized control trials (RCTs) found that participating in exercise programs reduces the rate of falls by 23% (high-certainty evidence). Balance and functional exercise programs reduce the rate of falls by 24% (high-certainty evidence), balance and functional exercises, and resistance exercises reduces the rate of falls by 34% (moderate-certainty evidence), while Tai Chi reduce the rate of falls by 19% (low-certainty evidence). There is uncertainty regarding whether resistance training, dance or walking prevents falls. Balance and functional exercises as well as Tai Chi mostly involve voluntary preplanned movements and maintaining balance during static postures or during movement balance. These types of exercises aimed to mainly to improve proactive balance response i.e., anticipatory postural adjustments i.e., APA's [4]. Many falls in older adults, occur when they fail to adequately respond to unexpected loss of balance i.e., unexpected perturbation [18]. A loss of balance can result from external perturbations, such as pushes, or when people fail to effectively maintain balance during voluntary movements i.e., self-induced internal perturbations, such as Tai Chi exercises. In both cases if balance is lost, a rapid and effective reactive balance response is required to prevent a fall. Therefore, specific training to improve the quality of reactions to a loss of balance induced by internal or external perturbations may be particularly effective for preventing falls [19–25]. Reactive Balance training (RBT), also known as perturbation-based balance training, i.e., PBBT, is a specific type of balance training where participants repeatedly experience unannounced balance loss, and need to execute balance reactions to avoid a fall [20]. Several previous studies have found that RBT can improve reactive balance control and prevent falls in daily life (19–25. In meta-analyses of RCT's, RBT was found to reduce the rate of falls by 40–46% compared to other exercise interventions [19–25]; this is almost twice the effect of ‘conventional’ balance and functional training, which include exercises that may or may not cause an unexpected loss of balance during the training sessions [15–17, 21]. In a recent meta-analysis, Devasahayam et al. [25] identified 14 RBT studies that monitored falls prospectively. Nine RBT studies used cumbersome equipment (e.g., perturbation treadmills), and 5 RBT studies used manual perturbation (e.g., lean-and-release or push/pull from therapist). The fall rate ratio of 6 perturbation treadmills studies was 0.43–0.85, suggesting that falls are less likely to occur in the RBT group. One study showed that falls are equally likely to occur in each group (fall rate ratio = 1). Two perturbation treadmills studies showed that falls are more likely to occur in the RBT group (fall rate ratio 1.11 and 1.41). Regarding the manual RBT studies, all five studies show that falls are less likely to occur in the RBT group (fall rate ratio 0.31–0.60). Thus, RBT methods where participants repeatedly experience external unannounced balance loss seem to simulate and closely mimic unexpected, real-life external perturbations for preventing falls in daily life. As such, it is an appropriate method that trigger and trains the body’s reactive balance control system to safely prevent falls. Although RBT has been shown to be effective as a fall-prevention program, the extent of its use in physical therapy (PT) clinics is unknown. Translating new knowledge into practice requires time and effort, and healthcare professionals experience barriers implementing new evidence-based practices [26]; therefore, it is likely that PTs experience challenges implementing RBT into practice. Our aim was to explore whether there is an evidence-practice gap for RBT and to determine what barriers and challenges delay implementing such RBT programs in PT clinics [27]. Mansfield et al. [28] explored those barriers and facilitators, and tried to determine the scope of RBT usage among clinicians in Canada. They found that over 75% of the respondents answered that they are using RBT. They also found that the most significant barrier preventing the use of RBT was the lack of knowledge about/and familiarity with RBT. Facilitators for RBT use were being able to complete RBT easily in the practice settings, having colleagues who use RBT, having access to resources for RBT, and having sufficient training and mastery of RBT. Replication of the Canadian study [28] in a different context is valuable since most of the resources available for RBT are published in English, so those who do not read English at all or read English as a second language would have difficulty accessing these resources. Also, differences in the academic studies and clinical rotations in different countries may drive clinical practice and decisions, and differences in the health-care systems in different countries may affect clinical practice.

Thus, the purpose of the current research was to estimate the extent of RBT use in PT clinics in Israel and to identify existing barriers to/ facilitators of implementing RBT in clinical practice among physical therapists, and to determine which aspects of RBT most interest physical therapists in Israel. Reactive balance training (RBT) in the present study was defined as a balance training method in which the patients experience loss of balance (or postural perturbation) purposely, in a way that requires them to use balance reactions in order to prevent falling. The participants are expected to improve their reactive balance control by exercising those balance reactions. In a RBT session, the perturbation can be internal (meaning the patient loses his balance while completing balance-demanding tasks) or due to external force (for example, a push or pull by the therapist or a device like a motorized treadmill). The key parts of perturbation training are: 1) the patients are purposely positioned in a situation that makes them lose their balance, 2) the aim is to improve their control over their reactive balance reactions, i.e., balance recovery skills. The collected data allows reliable and detailed analysis of the relationship between existing barriers and facilitators and the use of RBT among Israeli PT's. The identified factors potentially influencing the relationship may contribute to a better understanding the cross-sectional interactions and may help to develop a focused educational program that provides scientifically based rehabilitation program for balance control for nationwide use.

We expected that same barriers and facilitators would be found among the PT professionals in Israel vs. Canadian clinicians: 1) Although in a previous study about 75% of the Canadian cohort used RBT in their clinical practice we expect that due to the English barriers, RBT is used less frequent by the Israeli PT's (< 50% of respondents); 2) the lack of relevant knowledge and skill are the most prevalent barriers to the implementation of RBT; 3) having the relevant scientific knowledge and the ability to complete RBT easily at the workplace are the most prevalent facilitators for RBT implementation; and 4) physical therapists are most interested in learning the principles of practical RBT.

## Methods

### Participants

**Fig. 1:**
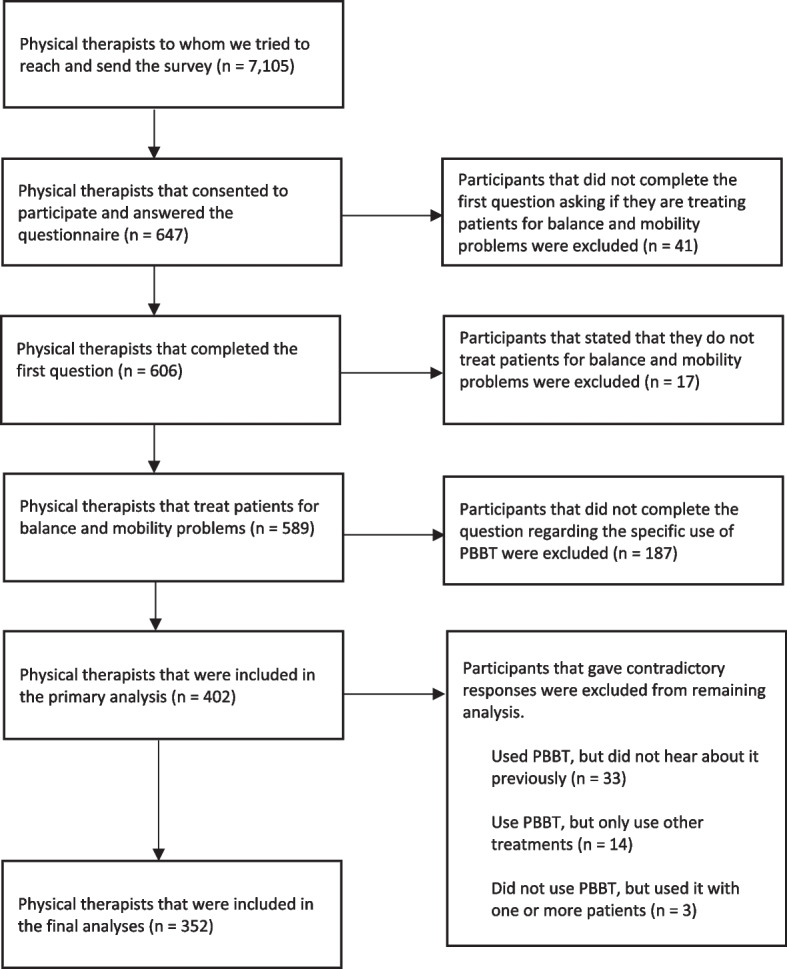
Survey's Flow Chart. The questionnaire was sent to 7,015 Physical therapists who are registered in the Israeli Physical Therapy Association (IPTS), 647 consented to participate, and 352 were included in the final analyses

In a cross-sectional survey we distributed via a link sent by Email with the help of the Israeli Physical Therapy Society (IPTS) to 7,105 registered Israeli physical therapists. The study was approved by the Ethics Committee of the Faculty of Health Sciences at Ben-Gurion University of the Negev (Ethics Committee approval number 38–2020). Only after indicating their consent by reading a consent form (using check mark), the participants filled out the questionnaire via Email and social networks. We included only registered PT's who stated that they practice clinical therapy intended to improve their patients’ balance or mobility. The inclusion and exclusion processes appear in Fig. 1.

### Questionnaire

We used an existing questionnaire developed by a Canadian team of investigators [28]. This questionnaire was developed based on a previous questionnaire [29]. A member of our research team translated the questionnaire into Hebrew (NM). Then, another team member, who had not seen the original questionnaire, translated it back into English (IK). Finally, a third member (IM) compared the original English version of the questionnaire to the retranslated English version, to assess its congruence, or lack of it. We did not find any major differences between the original questionnaire and the retranslated one. To ensure that all the items are culturally appropriate i.e., validate its content for Israeli physical therapists, the drafted questionnaire was given to a convenience sample of fifteen PT graduate students currently completing a master's degree at Ben-Gurion University of the Negev. They were tasked with critiquing the appropriateness of the individual items, to provide feedback on clarity and the overall survey length; the wording of some items was modified on the basis of this feedback. For example, one of the questions asked about RBT improving sensation, which can also be perceived as emotional feeling in Hebrew. We changed the term to clarify that we refer to physical rather than emotional sensation. All fifteen physical therapists agreed that the survey suited its purpose. The final questionnaire included a definition of RBT and five parts: 1) eligibility; 2) demographic information; 3) approaches for treating balance; 4) knowledge of RBT, including facilitators and barriers to its use; and 5) frequency of RBT use (see study questionnaire, supplementary material).

The translated questionnaire was sent as a digital link to registered Israeli physical therapists with the help of the IPTS. Prior to accessing the questionnaires, the participants were sent a Consent Affidavit, dealing with the research data, the researchers’ contact information, a privacy statement, and a statement confirming voluntary participation. The participants were required to click that they consent to participate to continue as part of this study. Submitted questionnaires were anonymous and accepted from October 2020 to April 2021; once a questionnaire was submitted, no further changes could be made. As instructed, the participants were not to put any personal, identifying information (i.e., name, address, etc.) on the questionnaires. After gathering all data from the questionnaires, the results were analyzed.

### Data and statistical analyses

Only those participants who met the eligibility criteria and had answered the questions regarding their general and specific use of RBT were included in the analysis. We calculated the descriptive statistics for each item in the questionnaire. We tested the first hypothesis by calculating the 95% confidence interval of the question regarding the general use of RBT. Respondents were then divided into three groups: 1) users (those who have either used RBT with more than one client to treat balance impairment, or use RBT regularly in their practice); 2) non-users (those who either never heard of RBT, or had previously heard about RBT, but were currently using other strategies to treat their clients’ balance impairments); and 3) open-to-use (those who know about RBT and are open to its use in their practice, but still have barriers to its practical implementation). The second and third hypotheses were tested by comparing the frequency of ‘agree’ and ‘disagree’ responses to the set of questions related to the barriers/facilitators experienced by the respondents at their workplace. Those answers were compared among the three groups using the chi-square test, and post-hoc pairwise comparisons were conducted using Fisher’s exact test. Additionally, we calculated the odds ratios (ORs) of chosen variables that showed significant differences between the users and the non-users in the post-hoc analysis. Those variables were categorized into three groups: 1) properties; 2) barriers; and 3) facilitators. We disproved tested between those variables by using the Spearman's rank correlation coefficient (r < 0.7) and calculated the ORs for each group by using multivariate logistic regression models. We also calculated the combined OR for each of the variables in the barriers group and in the facilitators group with univariate logistic regression models. Regarding the combined barriers, we took the variables from the barriers' multivariate logistic regression model and calculated the combined OR of an individual who has not experienced the following barriers: unsure of when to use RBT; it takes too much time to set-up for RBT; RBT is unsafe for patients; inability to conduct RBT without assistance. Regarding the combined facilitators, we took the variables from the facilitators' multivariate logistic regression model and calculated the combined OR of an individual who appreciates the following facilitators: ease of use of RBT at the clinic; encouraged by colleagues to use RBT; have managerial support and/or encouragement; and have access to necessary resources for using RBT). The fourth hypothesis was tested using the same methods as for the second and third hypotheses, regarding the questions on the learning needs around RBT. An alpha of 0.05 defined statistical significance in all the analyses.

## Results

### Participants

Six-hundred and forty-seven physical therapists consented to participate, filled out and submitted their questionnaires. Seventeen participants answered that they do not treat patients for balance or mobility problems, and forty-one participants did not answer this question; these 58 PT's were excluded from this study. One-hundred and eighty-seven participants did not complete the question regarding the specific use of RBT and so were also excluded. Therefore, responses from 352 participants were included in the analysis. The participants’ characteristics are presented in Table 1.

**Table 1:** Participant characteristics. Values presented as number of responses in each category, with percentage of the group in parentheses

	Non-users	Users	Open-to-use	Total
**Number**	59	217	76	352
**Gender**				
Male	16 (27.1)	69(31.8)	7(9.2)	92(26.1)
Female	43(72.9)	148(68.2)	69(90.8)	260(73.9)
**Highest level of education**				
Bachelor's	35(59.3)	122(56.5)	45(60)	202(57.7)
Professional master's	13(22)	41(19)	16(21.3)	70(20)
Thesis-based master's	10(16.9)	46(21.3)	13(17.3)	69(19.7)
PhD	1(1.7)	7(3.2)	1(1.3)	9(2.6)
Missing	0(0)	1(0.4)	1(1.3)	2(0.5)
**Professional experience**				
5 years or less	9(15.3)	43(19.8)	22(28.9)	74(21)
6–10 years	10(16.9)	40(18.4)	16(21.1)	66(18.8)
11–15 years	5(8.5)	38(17.5)	9(11.8)	52(14.8)
16–20 years	10(16.9)	14(6.5)	3(3.9)	27(7.7)
More than 20 years	25(42.4)	82(37.8)	26(34.2)	133(37.8)
**Time spent treating clients with balance problems (per week)**				
0–1 work day	23(40.4)	54(25.1)	15(19.7)	92(26.4)
1–2 work days	19(33.3)	89(41.4)	34(44.7)	142(40.8)
2–3 work days	8(14)	37(17.2)	17(22.4)	62(17.8)
3–4 work days	2(3.5)	25(11.6)	5(6.6)	32(9.2)
4–5 work days	5(8.8)	10(4.7)	5(6.6)	20(5.7)
Missing	2(3.5)	2(0.9)	0(0)	4(1.1)
**Healthcare setting**				
Acute care	11(19.3)	19(8.8)	9(11.8)	39(11.2)
Inpatient rehabilitation	7(12.3)	34(15.8)	13(17.1)	54(15.5)
Outpatient rehabilitation	1(1.8)	15(7)	2(2.6)	18(5.2)
Private practice	7(12.3)	21(9.8)	4(5.3)	32(9.2)
Home/community care	18(31.6)	73(34)	24(31.6)	115(33)
Long term care	3(5.3)	11(5.1)	8(10.5)	22(6.3)
Other	10(17.5)	42(19.5)	16(21.1)	68(19.5)
Missing	2(3.5)	2(0.9)	0(0)	4(1.1)
**Area of practice**				
Neurological	7(12.1)	53(24.8)	9(11.8)	69(19.8)
Orthopedic	26(44.8)	82(38.3)	34(44.7)	142(40.8)
Cardiorespiratory	1(1.7)	2(0.9)	0(0)	3(0.9)
Geriatric	7(12.1)	28(13.1)	15(19.7)	50(14.4)
Pediatric	7(12.1)	10(4.7)	7(9.2)	24(6.9)
Other	10(17.2)	39(18.2)	11(14.5)	60(17.2)
Missing	1(1.7)	3(1.4)	0(0)	4(1.1)

### Use of RBT

For clarity, the definition of RBT was included in the questionnaire (see the definition in the introduction), so that the respondents might be more confident when answering the yes/no question regarding their use of this method. Three hundred and three participants (out of 402), 75.4%, stated that they use RBT, with a 95% confidence interval (72.4, 77.8). Regarding its specific use, 11.2% of the participants (45/402) reported they had not heard about RBT before; and 15.2% of the participants (61/402) reported they had heard of RBT before, but are using other treatment options for balance and mobility dysfunction; 18.9% of the participants (76/402) stated they are familiar with RBT, but experience barriers when using this method in their practice; 39.6% (159/402) stated they had used RBT in their practice with more than one patient; and 15.2% (61/402) stated that they are using RBT on a regular basis.

Several participants had contradictions in responses to the yes/no question about RBT use and the specific use question; 33 participants responded that they had used RBT, but had not heard of it; 14 participants reported that they are using RBT, but stated that they are using other treatment strategies to treat balance and mobility problems; and 3 participants stated that they did not use RBT, but had used it with more than one patient. Those respondents with conflicting answers were removed from the analysis. The remaining 352 participants were divided into three groups, based on their responses to the specific use question: 1) user group (*n*=217); 2) non-user group (*n*=59); 3) open-to-use group (*n*=76). Treatment strategies for balance and mobility problems are shown in Table 2.

**Table 2:** Treatment strategies for balance and mobility problems. The values presented indicate the number of responses in each category, with percentage of the group in parentheses

	Non-users	Users	Open-to-use	Total
**Number**	59	217	76	352
**Client's admitting diagnosis**				
Stroke	32(54.2)	129(59.4)	40(52.6)	201(57.1)
Movement disorder including Parkinson disease	28(47.5)	131(60.4)	48(63.2)	207(58.8)
Spinal cord injury	7(11.9)	57(26.3)	19(25)	83(23.6)
Cerebral palsy	9(15.3)	31(14.3)	13(17.1)	53(15.1)
Acquired brain injury excluding stroke	10(16.9)	68(31.3)	14(18.4)	92(26.1)
Multiple sclerosis	7(11.9)	58(26.7)	15(19.7)	80(22.7)
Vestibular conditions	23(39)	68(31.3)	30(39.5)	121(34.4)
Dementia/cognitive impairment	19(32.2)	91(41.9)	33(43.4)	143(40.6)
Musculoskeletal conditions	43(72.9)	174(80.2)	59(77.6)	276(78.4)
COPD/respiratory conditions	12(20.3)	35(16.1)	16(21.1)	63(17.9)
Cardiac conditions	9(15.3)	34(15.7)	12(15.8)	55(15.6)
Geriatric	39(66.1)	152(70)	55(72.4)	246(69.9)
**Treatment strategies**				
Task-oriented training	37(62.7)	156(71.9)	55(72.4)	248(70.5)
Bobath/neurodevelopmental training	13(22)	66(30.4)	17(22.4)	96(27.3)
Overground walking practice	49(83.1)	177(81.6)	56(73.7)	282(80.1)
Body weight supported treadmill training	11(18.6)	56(25.8)	11(14.5)	78(22.2)
Functional electrical stimulation	1(1.7)	10(4.6)	1(1.3)	12(3.4)
Strength training	38(64.4)	158(72.8)	53(69.7)	249(70.7)
Aerobic/cardiorespiratory training	19(32.2)	92(42.4)	33(43.4)	144(40.9)
Reactive balance training	14(23.7)	185(85.3)	40(52.6)	239(67.9)
Video game-based interventions/exergaming	15(25.4)	72(33.2)	17(22.4)	104(29.5)
Specific exercise program (e.g., FaME, Otago etc.)	3(5.1)	24(11.1)	6(7.9)	33(9.4)

### Prior experiences, knowledge, and attitudes about RBT

Having received a RBT education in an entry-to-practice program was significantly different between the non-users’ and users’ groups, and between the non-users and the open-to-use group (*P*<0.001 and *P*=0.022, respectively, Table 3). The non-users were the least likely to have received RBT education in their entry-to-practice program, and there was no significant difference between the users’ group and the open-to-use group. In all the groups, most of participants stated they first heard about RBT during their bachelor’s studies. In all three groups, there was little agreement with the statement: “Tried RBT but found it to be ineffective;” with no statistically significant difference between the groups. The users were more likely than the non-users and the open-to-use group to agree that their patients acknowledged that RBT improved their balance (*P*=0.015 and *P*<0.001, respectively). Also, the users were more likely to state that they feel confident doing RBT, compared to the non-users and the open-to-use group (*P*<0.001 and *P*<0.001, respectively), and they were less likely to prefer other methods than RBT (*P*<0.001 and *P*<0.001, respectively). There was no statistically significant difference between the groups regarding whether they would like to use RBT more; 78.9%-92.4% of respondents in all the groups agreed with this statement. The non-users indicated that they are less familiar with RBT research, compared to the users’ group and open-to-use group (*P*<0.001 and *P*=0.012, respectively). There was no significant difference between the users and the open-to-use regarding their knowledge of scientific research evidence on RBT.

**Table 3:** Prior experiences, attitudes, and knowledge of RBT

	Non-users		Users		Open-to-use		
	n	Responses	n	Responses	n	Responses	*P*-value
**Prior experiences with RBT**							
Received RBT education in entry-to-practice program	58	17(29.3)	216	124(57.4)	75	37(49.3)	0.001^*†^
First heard about RBT							
Bachelor's degree	49	19(38.8)	213	90(42.3)	74	32(43.2)	0.005
Master's degree		3(6.1)		12(5.6)		8(10.8)	
Workplace/Colleagues		7(14.3)		52(24.4)		12(16.2)	
Continuing education		2(4.1)		32(15)		7(9.5)	
Physiotherapy conference		14(28.6)		17(8)		12(16.2)	
Physical therapist acquaintance		4(8.2)		10(4.7)		3(4.1)	
Tried RBT, but found it to be ineffective	39	1(2.6)	199	3(1.5)	64	2(3.1)	0.695
Clients acknowledge that balance improves with RBT	10	7(70)	157	150(95.5)	35	26(74.3)	< 0.001^†‡^
Confident in ability to conduct RBT with clients	46	22(47.8)	198	185(93.4)	63	30(47.6)	< 0.001^†‡^
Prefer to use other treatment options besides RBT	46	34(73.9)	177	72(40.7)	69	45(65.2)	< 0.001^†‡^
Would like to use RBT more	38	30(78.9)	191	157(82.2)	66	61(92.4)	0.096
Familiar with RBT research	58	15(25.9)	212	112(52.8)	73	35(47.9)	0.001^*†^
Research evidence that RBT can improve sensation	6	6(100)	51	44(86.3)	17	17(100)	0.175
Research evidence that RBT can improve walking function performance	8	8(100)	77	75(97.4)	23	22(95.7)	0.799
Research evidence that RBT can improve lower-limb muscle strength/endurance	5	5(100)	58	38(65.5)	18	16(88.9)	0.056
Research evidence that RBT can improve anticipatory balance control	8	8(100)	75	72(96)	22	20(90.9)	0.495
Research evidence that RBT can improve reactive balance control	8	8(100)	82	82(100)	23	23(100)	
Research evidence that RBT can improve coordination	4	4(100	57	53(93)	20	19(95)	0.827
Research evidence that RBT can improve spatial awareness	5	4(80)	60	56(93.3)	18	16(88.9)	0.528
Research evidence that RBT can reduce falls in daily life	8	8(100)	80	78(97.5)	24	24(100)	0.665
Research evidence that RBT can improve balance confidence	8	8(100)	79	78(98.7)	25	25(100)	0.810

(*P* < 0.0056 for nine pairwise comparisons) Only those who answered that they have practical knowledge about RBT were invited to answer questions regarding research evidence

^*^ Significant difference between the Non-user and Open-to-use groups (*P* < 0.0056 for nine pairwise comparisons)

^†^Significant difference between the Non-user and User groups (*P* < 0.0056 for nine pairwise comparisons)

^‡^ Significant difference between the Open-to-use and User groups

### Barriers to implementing RBT

The responses to the set of questions about barriers to the use of RBT were categorized and grouped into themes as follow: 1) practice setting, 2) equipment, 3) knowledge/training, 4) client characteristics, 5) time and 6) human resources. The answers to the set questions about barriers are presented in Table 4.

**Table 4:** Barriers to implementing RBT. Data presented are the total number of non-missing responses (n) and the number of responses in each category, for each item for each group, with the percentage of non-missing responses in parenthesis. The *p*-value is for the item chi-square test, comparing response rates between groups and categories of response. The symbols indicate results of pairwise comparisons

	Non-users		Users		Open-to-use		
	n	Responses	n	Responses	n	Responses	*P*-value
**Barriers related to practice setting**							
Limited space to set up equipment for RBT	32	24(75)	157	102(65)	52	38(73.1)	0.368
Clients' length of stay is too short to include RBT in treatment plans	35	14(40)	184	28(15.2)	64	17(26.6)	0.002^†^
**Barriers related to equipment**							
Do not have the authority to purchase equipment for RBT	41	23(56.1)	156	86(55.1)	56	43(76.8)	0.015^*‡^
Delivering effective manual perturbations can be fatiguing	35	16(45.7)	191	85(44.5)	64	37(57.8)	0.177
**Barriers related to knowledge/training**							
Unsure of when to use RBT	42	21(50)	202	40(19.8)	65	27(41.5)	<0.001^†‡^
**Barriers related to client characteristics**							
Weak evidence to justify the use of RBT with client population	31	7(22.6)	163	9(5.5)	51	4(7.8)	0.006^†^
Clients have communicated that they are apprehensive about RBT	26	10(38.5)	193	76(39.4)	57	27(47.4)	0.539
RBT is not safe for my clients	31	11(35.5)	190	13(6.8)	59	21(35.6)	<0.001^†‡^
Clients are too cognitively impaired to take part in RBT	36	4(11.1)	181	4(2.2)	60	5(8.3)	0.023^†‡^
**Barriers related to time**							
Takes too much time to set-up for RBT	25	13(52)	166	28(16.9)	44	16(36.4)	<0.001^†‡^
**Barriers related to human resources**							
Cannot conduct RBT without having someone else to assist	27	15(55.6)	173	32(18.5)	43	21(48.8)	<0.001^†‡^

^*^Significant difference between the Non-user and Open-to-use groups (*P* < 0.0056 for nine pairwise comparisons)

^†^Significant difference between the Non-user and User groups (*P* < 0.0056 for nine pairwise comparisons)

^‡^ Significant difference between the Open-to-use and User groups (*P* < 0.0056 for nine pairwise comparisons)

#### Practice setting

Most participants in all 3 groups (>50%) agreed that they have limited space in which to set up the RBT equipment. There was no significant difference between the groups regarding this item. The non-users were more likely than the users to agree that their clients’ length of stay is too short for conducting RBT (*P*=0.002).

#### Equipment

The open-to-use group were more likely than the users and non-users to state that they do not have the authority to purchase RBT equipment (*P*=0.004 and *P*=0.047, respectively). All three groups equally agreed with the statement that delivering effective manual perturbations can be fatiguing.

#### Knowledge/training

The users were significantly more likely to disagree about being unsure of when to use RBT than the non-users and the open-to-use (*P*<0.001 and *P*<0.001, respectively), with approximately 20% of participants acknowledging they are unsure of when to use this method. In the non-users’ group, half the participants stated they are unsure of when to use RBT and 41.5% of the open-to-use group agreed.

#### Client characteristics

Significantly more non-users stated that there is weak evidence to justify RBT use with their clients than in the users’ group (*P*=0.006). There was no significant difference among the groups regarding patients being apprehensive about RBT and, in all three groups, less than half of the physical therapists agreed with this statement. There were, however, significant differences between the users and the non-users, and also between the users and the open-to-use group, regarding RBT patient safety (*P*<0.001 and *P*<0.001, respectively). The majority of the users’ group (93.2%) disagreed that RBT is unsafe for their clients, while around one-third of the non-user physical therapists and the open-to-use group agreed with this statement. Moreover, fewer physical therapists in the users’ group stated that their client population is too cognitively impaired to practice RBT than among the non-users and in the open-to-use group (P=0.028 and P=0.045, respectively).

#### Time

The users were less likely than the non-users and the open-to-use to state that setting up RBT takes too much time, with only~17% agreement (*P*<0.001 and *P*<0.001, respectively). Fifty-two percent of the non-user physical therapists agreed with this statement, with no significant difference between non-users and open-to-use group.

#### Human resources

Similar results are seen regarding the human resources barriers. The users were less likely to state that they need assistance to do RBT, with only 18.5% agreeing with this statement, compared to~50% agreement in the non-users’ and open-to-use groups (*P*<0.001 and *P*<0.001, respectively).

### Facilitators for implementing RBT

Responses to set questions about facilitators for implementing RBT were categorized by themes as follow: 1) practice setting, 2) equipment, 3) knowledge/training. The answers to the set questions about facilitators are presented in Table 5.

**Table 5:** Facilitators for implementing RBT. Data presented are the total number of non-missing responses (n) and the number of responses in each category, for each item for each group, with the percentage of non-missing responses in parenthesis. The *p*-value is for the item chi-square test, comparing response rates between groups and categories of response. The symbols indicate results of pairwise comparisons

	Non-users		Users		Open-to-use		
	n	Responses	n	Responses	n	Responses	*P*-value
**Facilitators related to practice setting**							
Can easily complete RBT at place of practice	46	17(37)	205	187(91.2)	71	29(40.8)	<0.001^†‡^
One or more colleagues has used RBT	35	20(57.1)	164	159(97)	52	32(61.5)	<0.001^†‡^
Colleagues have encouraged use of RBT	39	6(15.4)	156	91(58.3)	59	17(28.8)	<0.001^†‡^
Manager supports and/or encourages use of RBT	26	9(34.6)	117	98(83.8)	35	18(51.4)	<0.001^†‡^
Purchasing equipment for RBT is within workplace budget	25	6(24)	132	76(57.6)	34	7(20.6)	<0.001^†‡^
**Facilitators related to equipment**							
Have access to resources necessary to use RBT	40	14(35)	173	96(55.5)	64	14(21.9)	<0.001^†‡^
**Facilitator related to knowledge/training**							
Have sufficient training in RBT	46	9(19.6)	174	89(51.1)	66	9(13.6)	<0.001^†‡^
With more hands-on training would be more inclined to use RBT	32	18(56.3)	162	88(54.3)	64	42(65.6)	0.299
Know what equipment is required to administer RBT	38	13(34.2)	167	99(59.3)	50	15(30)	<0.001^†‡^

^†^Significant difference between the Non-user and User groups (*P* < 0.0056 for nine pairwise comparisons)

^‡^ Significant difference between the Open-to-use and User groups (*P* < 0.0056 for nine pairwise comparisons)

#### Practice setting

The results show that the user physical therapists generally have more facilitators to implementing RBT, and a more supportive workplace environment. The users were more likely to report that they can easily complete RBT at their clinical practices with agreement rates of over 90%, while only about 40% of participants in the non-users’ group and in the open-to-use group claimed the same (*P*<0.001 and *P*<0.001, respectively). In all groups most physical therapists declared that they have one or more colleagues that use RBT, but there was a statistically significant difference in favor of the users’ group compared to non-users and the open-to-use group (*P*<0.001 and *P*<0.001, respectively). There was no significant difference between the non-users’ group and the open-to-use group regarding this matter. Results reveal that the users’ group also has more encouragement in their workplace; users were more encouraged by colleagues to use RBT than the non-users and the open-to-use groups (*P*<0.001 and *P*<0.001, respectively), with almost 60% of users agreeing that they received this encouragement, compared to 15% in the non-users’ group and 29% in the open-to-use group. The users’ group were also more likely to agree that they are encouraged by their manager compared to the non-users and the open-to-use group (*P*<0.001 and *P*<0.001, respectively), with almost 84% of agreement in the users’ group compared to the non-user's group (35%) and to the open-to-use group (31%). The users’ group was also more likely to agree that they have budget to purchase RBT equipment in their workplace compared to the non-users and to the open-to-use group (*P*=0.023 and *P*<0.001, respectively).

#### Equipment

More than half of the user participants stated that they have access to RBT resources and were significantly more likely to agree than 35% of the non-users’ group and 22% of the open-to-use group (*P*=0.023 and *P*<0.001, respectively).

#### Knowledge/training

Table 5 shows that the users’ group were more likely to agree that they have sufficient training in RBT than the non-users and the open-to-use group (*P*<0.001 and *P*<0.001, respectively) and that the users know what equipment is required to complete RBT, compared to the non-users and the open-to-use group (*P*=0.007 and *P*<0.001, respectively). No statistically significant difference was found between the non-users and the open-to-use group. Also, there was no statistically significant difference between all 3 groups regarding to the statement that with more hands-on training participants will be more inclined to use RBT. Note that there was a general agreement with the statement in the 3 groups.

### Odds ratios of properties, barriers, and facilitators

To understand the factors that influenced use of RBT, we calculated the OR using multivariate logistic regression models; we included variables that were statistically significant at the post-hoc pairwise comparisons of the users’ group and the non-user's group. We chose variables in which we had the most interest with and categorized them into three themes: 1) properties, 2) barriers, and 3) facilitators. After ruling out variable's collinearity, we created a multivariate logistic regression model for each theme. Variables and results for the properties, barriers and facilitators regression models are presented in Tables 6, 7 and 8 respectively.

**Table 6:** Users' properties, with a multivariate logistic regression model

	OR	95% CI	*p*-value
I have received education on RBT in my entry-to-practice healthcare-professional program	1.970	0.863–4.496	0.107
I am familiar with some research evidence surrounding RBT	1.162	0.501–2.696	0.726
I am confident in my ability to conduct RBT with my clients	11.363	4.402–29.331	< 0.001
I prefer to use other treatment options besides RBT	0.458	0.187–1.122	0.088

*OR* Odds ratio, represents the odds ratio to use RBT if agreeing with the matching sentence above. *CI* Confidence interval

#### Odds ratios of properties

Table 6 shows that individuals who agreed they have confidence in their ability to conduct RBT are 11.36 times more likely to be RBT users than those who declared they do not have confidence (95% CI=4.40, 29.33; *p*<0.001). Other variables did not attain statistical significance (Table 6).

#### Odds ratios of barriers

Physical therapists who agree that RBT is unsafe for their patients showed odds ratio of 0.130 to use RBT, suggesting that they are 7.69 times less likely to use RBT (1 / 0.130=7.69 times less likely) than those who disagree (95% CI=0.03, 0.52; *p*=0.004). Other variables did not reach statistical significance (Table 7).

**Table 7:** Users' barriers, with a multivariate logistic regression model

	OR	95% CI	*p*-value
I am unsure of when to use RBT in my practice	0.783	0.182–3.362	0.742
It takes too much time to set-up for RBT	0.345	0.092–1.288	0.113
RBT is not safe for my clients	0.130	0.033–0.521	0.004
I cannot conduct RBT with my clients without having someone else to assist me	0.393	0.110–1.412	0.152

*OR* Odds ratio, represents the odds ratio to use RBT if agreeing with the matching sentence above. *CI* Confidence interval

#### Odds ratios of facilitators

Considering facilitators, two variables attained statistical significance. Individuals who agree they can conduct RBT easily at their clinics are 41.53 times more likely to use RBT than those who disagree (95% CI=6.64, 259.97; *p*<0.001), and those who are encouraged by their colleagues to use RBT are 8.16 times more likely to be a RBT user than those who are not (95% CI=1.73, 38.49; *p*=0.008; Table 8).

**Table 8:** Users' facilitators, with a multivariate logistic regression model

	OR	95% CI	*p*-value
I can easily complete RBT at my place of practice	41.535	6.636–259.969	<0.001
My colleagues have encouraged me to use RBT	8.156	1.728–38.490	0.008
My manager supports and/or encourages use of RBT in my practice	2.692	0.526–13.767	0.234
I have access to resources necessary to use RBT in my practice	0.144	0.020–1.008	0.051

*OR* Odds ratio, represents the odds ratio to use RBT if agreeing with the matching sentence above. *CI* Confidence interval

### Odds ratios of combined barriers and combined facilitators

In addition to the OR of each of the chosen variables, we wanted to calculate the OR of the combined barriers and combined facilitators of the users’ group. We did two univariate logistic regression models, one for combined barriers and another for combined facilitators. For the combined barrier model, we calculated the OR for those individuals who met the following criteria: i) sure of when to use RBT; ii) disagree that it takes too much time to set-up for RBT; iii) disagree that RBT is unsafe for clients; and iv) disagree that they cannot conduct RBT without assistance. For the combined facilitator model, we calculated the OR for those individuals who met the following criteria: i) agreed they can easily do RBT at their clinics; ii) are encouraged by their colleagues to use RBT; iii) have their managers’ support and/or encouragement; and iv) have access to the necessary resources for using RBT. For each model, we calculated the ORs of those who met the above criteria. The results of the combined barrier and combined facilitator regression models appear in Table 9.

**Table 9:** Combined barrier and combined facilitator univariate logistic regression models

	OR	95% CI	*p*-value
Combined barriers	10.900	3.304–35.966	<0.001
Combined facilitators	4.572	1.060–19.722	0.042

*OR* Odds ratio, represents the odds ratio for the use of RBT, in accordance with the aforementioned criteria. *CI* Confidence interval

Combined barriers represent individuals that are sure of when to use RBT, disagree that it takes too much time to set-up for RBT, disagree that RBT is unsafe for clients, and disagree that they cannot conduct RBT without assistance. Combined facilitators represent individuals that agree they can easily do RBT at their clinics, are encouraged by colleagues to use RBT, have managerial support and/or encouragement, and have access to the necessary resources for doing RBT

Results show that those who meet the combined barrier criteria mentioned above are 10.9 times more likely to use RBT than those who do not meet them (95% CI=3.30, 36.00; *p*<0.001). Moreover, those who meet the combined facilitator criteria mentioned above are 4.5 times more likely to use RBT than those missing even one of those prerequisites (95% CI=1.06, 19.72; *p*=0.042). These results were statistically significant.

### Learning needs for promoting the use of RBT

The responses regarding preferred methods for learning about RBT were categorized by topics. Physical therapists stated whether, or not, they would like to know more about a certain topic and how they would prefer to learn (Table 10).

**Table 10:** Learning needs for promoting the use of RBT. The data presented are the total number of non-missing responses (n) and the number of responses in each category, for each item in each group, with the percentage of non-missing responses in parenthesis. The *p*-values represent each item in the chi-square test, comparing response rates among the three groups and the various response categories

	Non-users		Users		Open-to-use		
	n	Responses	n	Responses	n	Responses	*P*-value
**Interested** in** learning more about**							
Theoretical background of RBT	56	45(80.4)	206	172(83.5)	73	63(86.3)	0.664
Principles of training for RBT	58	53(91.4)	206	197(95.6)	74	68(91.9)	0.319
Identifying clients' reactive balance control impairments	56	50(89.3)	204	195(95.6)	73	69(94.5)	0.197
Specific approaches for clients' reactive balance control impairments	56	51(91.1)	206	196(95.1)	73	69(94.5)	0.503
**Interested in learning more about RBT through**							
'Hands on' workshop	49	38(77.6)	195	150(76.9)	70	54(77.1)	0.996
Reviewing case studies	45	30(66.7)	171	119(69.6)	65	46(70.8)	0.896
Having access to an expert to answer questions	47	36(76.6)	178	139(78.1)	66	52(78.8)	0.961
Reading scientific literature	46	35(76.1)	183	126(68.9)	64	46(71.9)	0.610
Reading training manual	46	35(76.1)	182	137(75.3)	67	57(85.1)	0.249
Watching instructional videos	55	51(92.7)	194	175(90.2)	75	70(93.3)	0.661
Webinar/teleconference	52	41(78.8)	180	135(75)	69	54(78.3)	0.778
An in-person lecture	50	32(64)	188	139(73.9)	63	46(73)	0.373

There were high rates of interest (80.4%-95.6%) in all the 3 groups, without significant differences between them indicating that most of the participating PTs (from all the groups) wanted more knowledge of/and familiarity with RBT. Among the users and non-users, most of the PTs answered that they would like to learn more about the principles of training for RBT, while in the open-to-use group, most of the PTs stated they are interested in learning more about identifying patients’ reactive balance control impairments and about specific approaches for treating such impairments. High interest (64%-93.3%) was also reported regarding the various learning options in PT education. In each group, the highest scores (90.2%-93.3%) were given for learning by means of watching instructional videos. Among the users, this choice was followed by attending practical ‘hands on’ workshops. The second choice of the open-to-use group was the reading of training manuals, while in the non-users’ group, it was followed by webinars/teleconferences.

## Discussion

Similar to the previous study with health-care professionals in Canada who use RBT [28], most of Israeli PTs who participated in the research report that they use RBT in their clinical practice (76.3% versus 75.4%). Also, in the Israeli cohort there were contradictions in responses, thus it is possible that some of the participants who declared using RBT are, in fact, not challenging reactive balance control, indicate a misunderstanding or confusion of the meaning of reactive balance control, although the definition was included in our questionnaire. It seems that participants in the Israeli cohort referred to an incidental loss of balance during practice rather than directed and specific training for reactive balance control. Interestingly, almost 40% of the PTs declared to have over 20 years of experience in their profession. This raises the possibility that these PT's have a limited knowledge regarding RBT. The RBT is clinically evident to prevent falls and to improve reactive balance control only around the past 10–15 years [23, 30, 31], thus it is possible that time from graduation is a factor that may influence their response.

We noticed that barriers related to practice setting (i.e., the limited space to set up equipment) and not having the authority to purchase RBT equipment were the most prevalent, indicating that PTs may falsely believe that RBT requires large and expansive equipment. Interestingly, Aviles et al. [32] reported that retirement community residents who participated in RBT also identified the cost of equipment as a main barrier related to RBT intervention. This is not always the case since perturbations can be acquired with simple and small equipment such as 'balance Swiss ball' exercises or step training [33–35]. For example, due to the multi-link structure of the human body, placing older adults in unsteady situations using balance Swiss and flat balls, will impose a perturbation of posture which require continuous postural adjustments, and may cause balance loss, which may trigger balance reaction to prevent falling. It was found that these types of exercises [33–35] improve proactive balance skills but the investigators did not monitor falls post training. We also found that the non-users were the least likely to receive RBT education in their entry-to-practice program, and the least familiar with RBT research studies compare to other groups. The non-users were also less sure than the users of when to use RBT, indicating on a gap in knowledge between these two groups. These findings are similar to the results of Mansfield et al. [28], who found lack of knowledge to be the most significant barrier for those who do not use RBT. Other interesting findings were related to the patients' properties. The non-users’ group seemed to have more patients than the users’ group who were reportedly too cognitively impaired to treat with RBT. This finding fits with the multivariate logistic regression model that show that the non-users’ group tended to believe more than the users’ group that RBT is unsafe for their patients, although there was no difference regarding thinking their patients are apprehensive of RBT. Additionally, compared to users, the non-users also tended to agree that their patients do not stay long enough in order to implement RBT.

Regarding the facilitators to implement RBT the most frequent facilitator was having at least one PT colleague that uses RBT. Generally, the users group seemed to have more supportive workplace environment to use RBT since they were more likely to have a user colleague, had more encouragement from their colleagues, and had more encouragement from their manager to use RBT. Other facilitators were access to resources, budget for equipment, sufficient RBT training, knowledge of required equipment and being able to easily complete RBT in practice. These results match with previous research that tried to identify facilitators to implement rehabilitation methods in practice [28, 29].

Similarly to the Canadian cohort [28] the majority of PTs in our cohort reported high interest to learn all the different topics of RBT. The two topics that garnered the most interest was identifying patients’ reactive balance control impairments, specific approaches for treating such impairments and to know more about principles of RBT training. The most preferred way to increase knowledge and familiarity with RBT was by watching instructional videos, which was also the most favored way in the Canadian research [28].

The current study has several limitations; first, we used a questionnaire that was distributed via several channels, such as Email and social networks. Using the format of a ‘convenient study’ enabled us to reach a relatively large number of PTs, but there is an accompanying risk of incurring bias. In our sample the entire population of PTs were eligible to participate, but it might be that those PTs who chose to respond were more interested in RBT. Also, there was a possibility that only physical therapists who think they use RBT would complete our questionnaires, for example, the 187 PTs who did not complete the questionnaire regarding the use of RBT. This may result in an over-representation of PTs reporting the use of RBT in their clinical practices (75.4%) and skewing the results, as if more PTs are using RBT than actually do so and thus, are likely not capturing the perspectives of those who have the most challenges or less interest with RBT. Another limitation is the lack of information on the type of RBT used by PTs in this study. Knowledge of the type of RBT used, i.e., large and cumbersome equipment or manual perturbations, may help better understand the PTs knowledge and barriers regarding RBT. We also considered the possibility that some PTs might feel as if they are being evaluated/examined and, as such, might provide insincere responses. To avoid such imprecisions, we emphasized that the survey is anonymous, hoping that the participants would feel more comfortable giving us honest answers. Another limitation was discovered in some of the answers themselves; there were several internal contradictions in PTs’ responses to certain questions, perhaps suggesting a misunderstanding of ‘reactive balance control’. In an attempt to overcome such contradictions, we excluded those responders from the affected analysis; a similar observation was made by Mansfield et al. [28] raising the possibility that this misunderstanding is quite common. This possible confusion regarding ‘reactive balance control’ and the false beliefs regarding RBT (such as the necessity for the use of large and cumbersome equipment), had led us to conclude that some misconceptions still surround this topic.

In conclusion, continued effort is necessary to increase the implementation of RBT in practice. The results suggest that there are misconceptions and insufficient knowledge about RBT among physical therapists, indicating that they categorize RBT as external perturbation training only. We believe that there is a need to improve clinicians’ knowledge and familiarity regarding RBT therapy, in order to widely implement this highly effective method for preventing falls in the older population in Israel and worldwide. Creating reliable information sources for RBT should help to reduce such doubt and hesitation by expanding the physical therapists’ practical knowledge, along with facilitating the implementation of instructional and therapeutic programs that will include both, self-induced internal perturbation exercises as well as external perturbation exercises, which hopefully reduce the risk of future falls and their costly consequences.

### Supplementary Information

**Additional file 1.** A survey of experience of physiotherapists in Israel using reactive balance training.

## Data Availability

The datasets used and/or analyzed in the current study are available from the corresponding author upon reasonable request.

## Abbreviations

CI: Confidence Interval
IPTS: Israeli Physical Therapy Society
OR: Odds ratios
RBT: Reactive balance training
PT: Physical Therapy
RCT: Randomized Control Trials
