# Supplementary material for: A survey of Israeli physical therapists regarding reactive balance training
Source: BMC Geriatr. 2023 Oct 13;23:656. doi: 10.1186/s12877-023-04356-5 (PMC10571354; doi:10.1186/s12877-023-04356-5)
Supplement: Supplementary file 1 — Additional file 1. A survey of experience of physiotherapists in Israel using reactive balance training. [file 12877_2023_4356_MOESM1_ESM.docx]

**A survey of experience of physiotherapists in Israel using reactive balance training**

This survey is written in feminine gender but addresses both genders.

**Informed consent**

We invite you to take part in a research titled "A survey of experience of physiotherapists in Israel using reactive balance training". The purpose of this research is to study the practical experience in using balance training in Israel. The research is led by Noam Margalit (a master's student at Ben Gurion university) and supervised by Dr. Ilan Kurz and Prof. Itshak Melzer from the department of physical therapy at the Ben Gurion University. This survey was sent to you since you are a physiotherapist working in Israel (in either a health organization or privet). The survey will collect demographic data as well as data regarding using and implementing balance training in your practice and regarding assistive or obstructive factors using balance training. Completing the survey (using the link below) will take 10-20 minutes of your time.

We believe there are no known hazards to taking part in this study. Your answers will remain confidential. Please do not provide any information that might identify you (such as your name, telephone number or address) within your answers. Your answers will remain anonymous and will be kept on safe servers of the Ben Gurion University, available only to the researchers. The data will be kept seven years from the end of this study and would then be deleted. You will not directly gain from your participation in the study, however we hope your participation will contribute to the collective effort of improving balance training in the future.

Taking part in the study is voluntary, we will be glad if you complete all of it but you choose to skip certain questions or stop at any time with no consequences to you. The survey is anonymous, meaning your answers will be sent to us using the link and we cannot trace the source, do to this it is important that you don’t include any personal information that might identify you. If you choose to take part in the study by replying to the survey and sending it and from whatever reason decide later on to quit, we might not be able to retract your answers do to the anonymity of the survey.

If you have questions regarding this project you can contact researcher Noam Margalit, phone number 052-8074605. If you have any questions regarding your rights as a participant you can also contact Dr. Ilan Kurz (Tel. 052-5013905) and Prof. Itshak Melzer (Tel. 050-8807990) from the department of physical therapy at the Ben Gurion University. Any discussions will remain confidential.

Your agreement to take part in the study is implied from completing the survey and sending it using the link. If you are not interested in taking part in the study please do not complete or send back the survey.

**Part A: training**

1. **My primary training is:**

- A physiotherapist
- A kinesiologist
- Other:______________
- I am not a professional care provider

1. **I provide direct clinical treatment aimed at improving the balance/ mobility of my patients and/or prevent them from falling in their daily life.**

- Yes
- No

**If you answered that you are not a professional care provider in question1 or "No" in question 2, please do not proceed with the survey. Only professional care providers that provide clinical treatment aimed at improving the balance/ mobility of their patients and/or prevent them from falling in their daily life are suited for this study.**

**Part B: You and your work**

1. **I identify myself as:**
   - Male
   - Female
   - Non binary/ third gender
   - I would rather describe myself as:________
   - I rather not answer
2. **My age is:**
   - ≤30
   - 31-40
   - 41-50
   - 51-60
   - 61-70
   - ≥70
3. **Level of education acquired (please mark al the relevants)**
   - A diploma
   - First degree (BSc for example)
   - Second degree
   - Doctorate (DPT for example)
   - Doctorate with thesis (PhD)
4. **I work as a professional care giver for:**
   - ≤5 years
   - 6-10 years
   - 11-15 years
   - 16-20 years
   - ≥20 years
5. **I work mainly at:**
   - Galil and Golan regions
   - Haiffa and the north coast region
   - The valeys (yisrael, hamesholash up to Hedera)
   - Central coast region and Sharon
   - Dan district
   - South coast region
   - South and Negev area
   - Eilat
6. **The population of the town I work in is (if you are not sure what you believe is the closest):**
   - ≤10000
   - 10000-50000
   - 50000-100000
   - 100000-250000
   - ≥250000
7. **The amount of time I devote, during a normal week, to direct clinical care aimed at improving my patients balance/ mobility or prevent them from falling in their daily life is:**
   - 0-7.5 hours (0-1 hours a day)
   - 8-15 hours (1-2 hours a day)
   - 15.5-22.5 hours (2-3 hours a day)
   - 23-30 hours (3-4 hours a day)
   - 30.5-37.5 hours (4-5 hours a day)

Please notice, if you have several duties and/or work in more than one place, please answer the rest of the questions in this part (10-13) with regard to the job where you provide direct clinical care aimed to improve balance/ mobility of your patients, and/or prevent them from falling in their daily life.

1. **My work setting which provides the most balance training/ fall prevention treatment is:**
   - Intensive care
   - Inpatient rehabilitation
   - Outpatient rehabilitation
   - Privet clinic
   - Home/ community care
   - Long term day care
   - Other (please detail):__________
2. **My scope of practice in the work setting I chose in question 10 is:**
   - Neurologic
   - Orthopedic
   - Cardio-respiratory
   - Geriatric
   - Pediatric
   - Other (please detail):_________
3. **The age group of my patients in the work setting I chose in question 10 is:**
   - ˂18
   - ≥18
   - All ages
4. **The work setting I chose in question 10 is considered a university hospital (meaning it is fully associated to a university)**
   - Yes
   - No
   - I don’t know/ not sure

**Part C: Regarding your treatment attitude**

1. **The patients I treat for balance/ mobility problems are people who were diagnosed with (mark all that are relevant):**
   - Stroke
   - Parkinson and/or other movement disorders
   - Spinal cord injury
   - Cerebral palsy
   - Head injury which is not stroke
   - Multiple sclerosis
   - Vestibular dysfunction
   - Dementia
   - Musculoskeletal problems
   - COPD or respiratory problems
   - Cardiac dysfunctions
   - Complicated geriatric patients
   - Other neurologic syndromes, please detail:_______
   - Other, please detail:_________
2. **I currently use the following treatment strategies to treat balance/mobility dysfunctions of my patients and/or for daily fall prevention (mark all the relevant):**
   - Task specific training
   - Bobath/ neurodevelopmental training
   - Over ground walking training
   - Body-weight supported treadmill training
   - Functional electric stimulation
   - Strength training
   - Cardio-respiratory aerobic training
   - Perturbation based balance training
   - Video games based interventions/ exergaming (for example Wii balance plate)
   - Specific balance training (for example, FAME, Otago) please detail:__________________________
   - Other, Please detail:______________

Please answer all the questions in parts A-C that you meant to answer before you continue to the next part. **Please don’t change your answers to parts A-C after starting part D.**

**Part D: Perturbation based balance training**

Perturbation based balance training or PBBT is a balance training method in which the patients experience loss of balance (or postural perturbation) purposely, in a way that requires them to use balance reactions in order to prevent falling. By exercising those balance reactions over, the participants are expected to improve their reactive balance control. In a PBBT session the perturbation can be internal (meaning, the patient loses his balance while completing balance demanding tasks) or do to external force (for example, a push or pull by the therapist or a device like a computerized treadmill).

The key parts of a perturbation training are: 1) the patients are purposely positioned in a situation that makes them lose their balance, 2) the aim is to improve their control over their reactive balance reactions (for example, balance recovery skills).

1. **Given the definition of PBBT above, I used PBBT in my clinical practice**
   - Yes
   - No
2. **The following statement describes me the best with regards to PBBT (choose only one):**
   - I didn't hear of PBBT until now
   - I have heard of PBBT before, but currently I am using different strategies to address my patients balance problems.
   - I know about PBBT and open to use it in my clinic, but I experience difficulties/ barriers implementing it in my clinic.
   - I have implemented PBBT with more than one of my patients to address balance problems.
   - I use PBBT regularly in my clinic (meaning, with at least half of my patients who has reactive balance dyscontrol).
3. **I received education regarding PBBT in study program**
   - Yes
   - No

1. **I was first exposed to PBBT through (use your own words):_______________________________________________________**
2. **How much do you agree or disagree with the following statements regarding your experience treating patients with balance/ mobility problems?**

|  | **Do not agree at all** | **Do not agree** | **Agree** | **Highly agree** | **Not sure** | **Not relevant** |
| --- | --- | --- | --- | --- | --- | --- |
| 1. **I can easily complete/ preform PBBT in my practice** |  |  |  |  |  |  |
| 1. **I have confidence in my abilities to perform PBBT training with my patients** |  |  |  |  |  |  |
| 1. **I rather use different treatment options than PBBT** |  |  |  |  |  |  |
| 1. **The evidence is too weak to justify using PBBT with my patients** |  |  |  |  |  |  |
| 1. **I have sufficient training in the field of PBBT** |  |  |  |  |  |  |
| 1. **As a therapist, giving effective manual perturbations could be tiring** |  |  |  |  |  |  |
| 1. **If had more hands on sessions I would be more inclined to use PBBT exercises** |  |  |  |  |  |  |
| 1. **I am not sure when I should use PBBT in my work** |  |  |  |  |  |  |
| 1. **I have tried PBBT in the past , but found it inefficient** |  |  |  |  |  |  |
| 1. **I would like to use PBBT more** |  |  |  |  |  |  |
| 1. **My patients reported they are afraid of PBBT** |  |  |  |  |  |  |
| 1. **To the best of my knowledge one or more of my colleges have used PBBT in their clinical practice** |  |  |  |  |  |  |
| 1. **My colleges have encouraged me to use PBBT** |  |  |  |  |  |  |
| 1. **I have access to the resources needed to use PBBT in my clinical practice** |  |  |  |  |  |  |
| 1. **I don’t have the authority to perches the equipment needed for PBBT** |  |  |  |  |  |  |
| 1. **Where I work purchasing PBBT equipment is in the budget** |  |  |  |  |  |  |
| 1. **We have limited space for the equipment needed for PBBT** |  |  |  |  |  |  |
| 1. **I know what is the equipment needed to preform PBBT** |  |  |  |  |  |  |
| 1. **The visit time of my patients is too short to include PBBT in their treatment plan** |  |  |  |  |  |  |
| 1. **It takes too long to prepare for PBBT** |  |  |  |  |  |  |
| 1. **PBBT is not safe for my patients** |  |  |  |  |  |  |
| 1. **My supervisor supports and/ or encourages the use of PBBT where I work** |  |  |  |  |  |  |
| 1. **I cannot practice PBBT with my patients without the help of another person** |  |  |  |  |  |  |
| 1. **When I perform PBBT some of my patients acknowledge that their balance is improving** |  |  |  |  |  |  |
| 1. **My patients are too cognitively impaired to perform PBBT** |  |  |  |  |  |  |
| 1. **I have the opportunity to perform PBBT at my work** |  |  |  |  |  |  |

1. **The following things prevented me from using PBBT at my work (please live empty if nothing have prevented you from using PBBT at work):_______________________________________________________________________________________________________________________________________________________________________________________**
2. **The following things helped me to use PBBT at my work (please live empty if nothing helped you to use PBBT at work): _____________________________________________________________________________________________________________________________________________________________________________________________**
3. **I know some research evidence regarding PBBT**
   - Yes
   - No
4. **If you answered "yes" to question 23, please mark if you agree or dis agree with the following statements:**

|  | **Agree** | **Disagree** | **Don’t know** |
| --- | --- | --- | --- |
| 1. **There is research evidence that PBBT can improve sensation** |  |  |  |
| 1. **There is research evidence that PBBT can improve function/ walking** |  |  |  |
| 1. **There is research evidence that PBBT can improve power/ endurance of lower limb muscles** |  |  |  |
| 1. **There is research evidence that PBBT can improve anticipatory balance control** |  |  |  |
| 1. **There is research evidence that PBBT can improve reactive balance control** |  |  |  |
| 1. **There is research evidence that PBBT can improve coordination** |  |  |  |
| 1. **There is research evidence that PBBT can improve patient's special awareness** |  |  |  |
| 1. **There is research evidence that PBBT can decrease falls in daily life** |  |  |  |
| 1. **There is research evidence that PBBT can improve balance self confidence** |  |  |  |

1. **As a health provider providing or the thought of providing PBBT makes me feel (please mark all the relevanta):**
   - Fear
   - Stress
   - Optimistic
   - Pessimistic
   - Excited
   - Sad
   - Uncomfortable
   - Comfortable
   - Calm
   - Encouraged
   - None of the above
2. **I would like to study more about PBBT through:**

|  | **Yes** | **No** |
| --- | --- | --- |
| 1. A Hands on course |  |  |
| 1. Review of case studies |  |  |
| 1. Access to an expert that can answer my questions |  |  |
| 1. Reading scientific literature |  |  |
| 1. Reading a training manual |  |  |
| 1. Watching video tutorials |  |  |
| 1. On-line seminar/ conference call |  |  |
| 1. A lecture personally attended |  |  |
| 1. None of the above |  |  |

**If you answered "yes" in question 16 please continue to part E. if you answered "No" in question 16 please continue to part F.**

**Part E: using Perturbation Based Training (PBBT) in your privet practice**

1. **I use perturbation based training to address the following dysfunctions or targets with my patients:**

|  | **Most of the time**  **(≥80%)** | **Often**  **(60-79%)** | **Sometimes**  **(41-59%)** | **Occasionally**  **(21-40%)** | **Rarely**  **(1-20%)** | **Never**  **(0%)** |
| --- | --- | --- | --- | --- | --- | --- |
| **Sensation improvement** |  |  |  |  |  |  |
| **Improving walking performance/ function** |  |  |  |  |  |  |
| **Improving muscle power/ endurance** |  |  |  |  |  |  |
| **Improving proactive (anticipatory) balance control** |  |  |  |  |  |  |
| **Improving reactive balance control** |  |  |  |  |  |  |
| **Improving coordination** |  |  |  |  |  |  |
| **Improving special awareness** |  |  |  |  |  |  |
| **Decreasing daily falls** |  |  |  |  |  |  |
| **Improving balance self confidence** |  |  |  |  |  |  |

1. **I use PBBT for something else other than the options above**
   - Yes, please detail:_______________________________________
   - No
2. **The following assessment tools help me to devise a PBBT treatment plan for my patients (write all those you find beneficial):___________________________________________________________________________________________________________________________________________________________________________________**
3. **Where I work I usually treat each patient:**
   - Less than once a week
   - Once a week
   - 2-3 times a week
   - 4-5 times a week or every day

**Usually the PBBT treatment program I give to my patient would be:**

1. **Usually I will do PBBT session with my patient:**
   - Less than once a week
   - Once a week
   - 2-3 times a week
   - 4-5 times a week or every day
2. **Usually the PBBT session would last:**
   - ≤15 minutes each time
   - 15-30 minutes each time
   - 31-45 minutes each time
   - ≥45 minutes each time
3. **Usually PBBT program would last**
   - ≤3 weeks
   - 4-6 weeks
   - ≥6 weeks

**Part F: future contact**

**If you are interested that we would contact you in order to take part in future research in this subject/ sharing the results of the current study, please let us know by sharing your e-mail.**
